# Decreasing auditory input induces neurogenesis impairment in the hippocampus

**DOI:** 10.1038/s41598-020-80218-z

**Published:** 2021-01-11

**Authors:** Takaomi Kurioka, Sachiyo Mogi, Taku Yamashita

**Affiliations:** Department of Otorhinolaryngology, Head and Neck Surgery, Kitasato University, 1-15-1 Kitasato, Minami-ku, Sagamihara-shi, Kanagawa 252-0374 Japan

**Keywords:** Auditory system, Cochlea, Adult neurogenesis

## Abstract

Hearing loss is associated with cognitive decline and dementia risk. Sensorineural hearing loss suppresses hippocampal neurogenesis, resulting in cognitive decline. However, the underlying mechanism of impaired neurogenesis and the role of microglial activation and stress responses related to hearing loss in the hippocampus remains unknown. Using a conductive hearing loss (CHL) model, we investigated whether a decrease in sound level could induce impairment of hippocampal neurogenesis and examined the differences between unilateral CHL (uCHL) and bilateral CHL (bCHL). To establish the CHL mouse model, ears were unilaterally or bilaterally occluded for five weeks by auditory canal ligation. Although hearing thresholds were significantly increased following CHL, CHL mice exhibited no significant loss of spiral ganglion or hippocampal neurons. Hippocampal neurogenesis was significantly and equally decreased in both sides following uCHL. More severe decreases in hippocampal neurogenesis were observed in both sides in bCHL mice compared with that in uCHL mice. Furthermore, microglial invasion significantly increased following CHL. Serum cortisol levels, which indicate stress response, significantly increased following bCHL. Therefore, auditory deprivation could lead to increased microglial invasion and stress responses and might be a risk factor for hippocampal neurogenesis impairment.

## Introduction

Aging is one of the key factors significantly influencing brain function impairment, including cognitive decline^1^. As elderly populations increase, maintaining better brain function, including cognitive performance, is expected to be critical for a good quality of life. Specifically, the hippocampus plays important roles in cognition, learning, and memory functions and is a major brain region shown to exhibit the capacity of neurogenesis. Newly generated neurons are thought to contribute to hippocampus-dependent cognitive functions^2,3^. In the process of hippocampal neurogenesis, microglial cells, the resident macrophages of the brain, are an important element for coordinating inflammatory responses in the brain^4^. Microglial activation has been reported to be increased in patients with cognitive impairment compared to age-matched controls, indicating that neuroinflammation-derived microglia may contribute to cognitive decline^5,6^. Furthermore, microglial activation is influenced by stress responses that also lead to changes in hippocampal neurogenesis, indicating that microglial activation and stress responses are important for regulating hippocampal neurogenesis^7^.

Recently, increasing evidence seems to support a relationship between hearing loss (HL) and cognitive decline in the elderly^8,9^. In research using animal models, sensorineural hearing loss (SHL) induced by noise overexposure suppressed hippocampal neurogenesis, and the animals exhibited cognitive decline^10,11^. Furthermore, cochlear lesions induced microglial activation and interactions between microglia and damaged auditory neurons in the central auditory system^12^. However, the mechanisms of hippocampal neurogenesis impairment and the role of microglial activation and stress responses following HL are not well understood.

Conductive hearing loss (CHL) has also been shown to alter the auditory system^13,14^. Decreased efficiency of sound transmission due to external or middle ear disease, without primary neuronal loss in the more central region, is the typical pathophysiology of CHL^15^. However, we recently reported on the cochlear synaptopathy and central synaptic alterations resulting in the cochlear nucleus after CHL^14,16^. This result indicates that CHL, decreasing the sound levels, could cause changes to neural components in peripheral and central auditory system. Despite being the second most clinically prevalent form of HL, the effects of CHL on hippocampus neurogenesis and cognitive function have received little attention. Furthermore, not all CHL patients receive medical treatment, especially those with unilateral CHL, due to the compensation mechanism of maximization of their better ear. However, it is not clear whether leaving CHL for a long time without medical treatment affects not only hearing but also brain function. Thus, elucidating whether the decreased auditory inputs alone, without neuronal loss, result in impairment of hippocampal neurogenesis is important for choosing the appropriate medical treatment for CHL patients.

In the current study, we investigated whether decreased sound levels without primary neuronal loss were sufficient to cause neurogenesis impairment and microglial activation in the hippocampus. To this end, we established an animal model of CHL by unilaterally or bilaterally ligating mice ear canals and examined the expression of markers of neurogenesis and microglial cells. Our data suggest that auditory deprivation could lead to increased microglial invasion and stress responses, and finally impair hippocampal neurogenesis.

## Results

**Figure 1:**
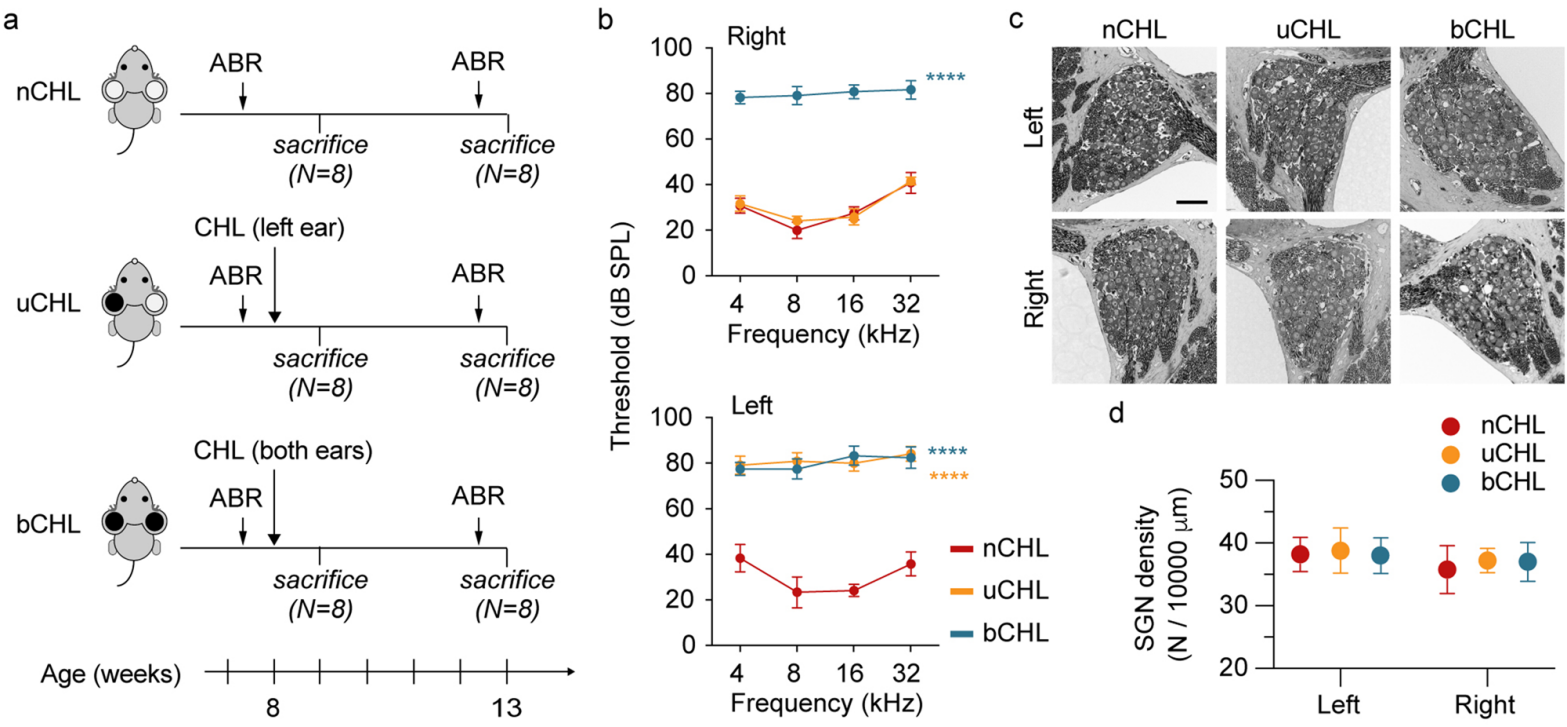
Experimental schedule and effects of ear occlusion on hearing thresholds and SGN survival. (**a**) Experimental schedule. (**b**) ABR thresholds at 5-weeks post occlusion (5 animals per group). Both ears in bCHL mice and the left ear in uCHL mice exhibited dramatic increases in ABR thresholds. ****p < 0.0001. (**c**) SGNs were densely packed in all groups. Scale bar indicates 50 μm. (**d**) Quantitative assessment of SGN density demonstrated that there were no significant differences among groups of mice in any ears (three animals per group). ABR, auditory brainstem response; SPL, sound pressure level.

In this study, 8-week-old male C57BL/6 mice were used to perform the ear occlusions by earplug insertion and auditory canal ligation. Animals were divided into three groups (Fig. 1a). The groups included mice with no occlusion (nCHL; n = 16 animals), mice with unilateral occlusion (uCHL) of the left ear for five weeks (n = 16 animals), and mice with bilateral occlusion (bCHL) for five weeks (n = 16 animals). The resulting uCHL and bCHL mice exhibited comparable appearance to nCHL mice and no abnormal behavior without HL. No mice experienced re-opening of auditory canals after initial ligation requiring re-ligation.

### Hearing assessment and spiral ganglion neuron survival

Baseline auditory brainstem response (ABR) thresholds were measured at 8-weeks of age, prior to ear occlusion. No significant differences in ABR thresholds among groups were observed at baseline. Ears were then occluded by plugging and ligating the auditory canal in the left ear for the uCHL group and in both ears for the bCHL group. To evaluate the influence of ear occlusion on hearing, ABR thresholds were measured at 5-weeks post occlusion. Dramatic increases in ABR thresholds were observed in the occluded ears at all frequencies (Fig. 1b). ABR thresholds were significantly higher in the left ear of uCHL mice and both ears of bCHL mice compared to those in either ear of nCHL mice (two-way analysis of variance (ANOVA), *p* < 0.0001). There were no significant differences in ABR thresholds between the right ear of uCHL mice and either ear of nCHL mice (two-way ANOVA, *p* = 0.84; Fig. 1b). To examine the degeneration of auditory neurons after CHL, we assessed the number of spiral ganglion neurons (SGNs) in each group of mice (n = 3 animals per group; Fig. 1c). No significant differences were observed in SGN density among groups (left side, one-way ANOVA, *F*_(2,12)_ = 0.02, *p* = 0.98; right side, one-way ANOVA, *F*_(2,12)_ = 0.06, *p* = 0.94; Fig. 1d), indicating that ear occlusion had no impact on cochlear neuronal survival.

### Hippocampal neuron survival

**Figure 2:**
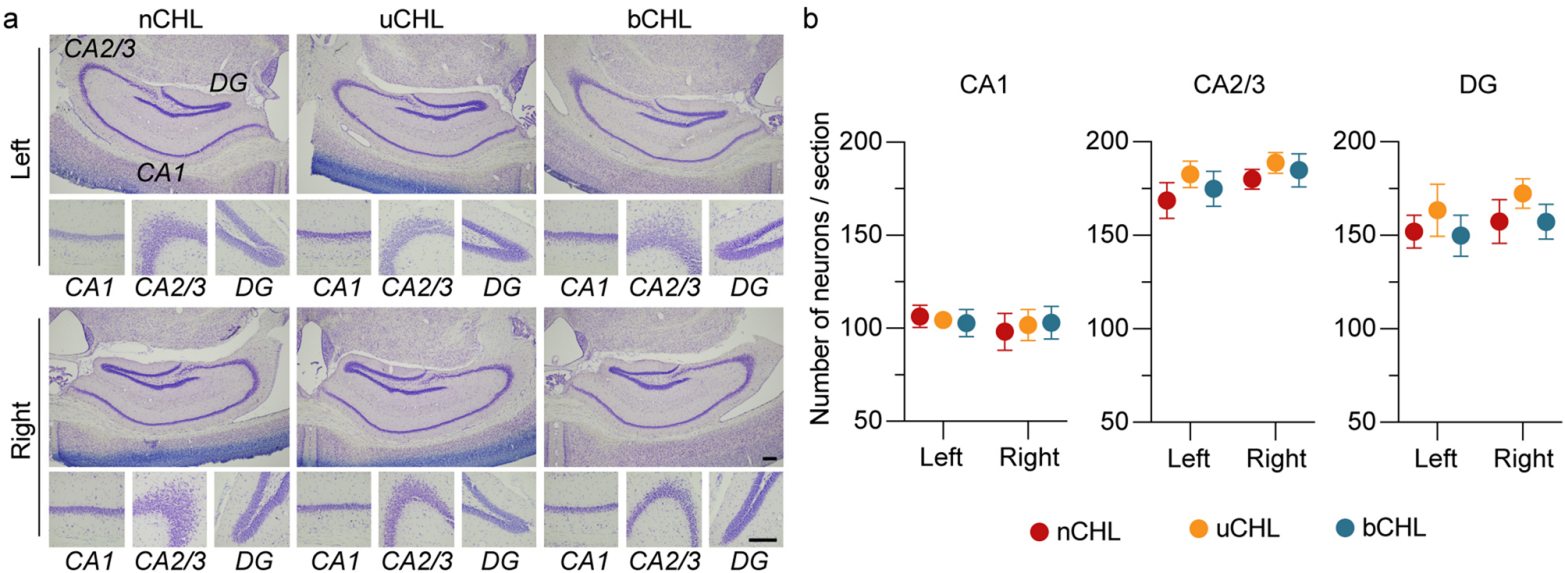
Neuronal density in the hippocampus showed no significant change after auditory deprivation. (**a**) Representative images of CA1, CA2/3, and DG regions of the hippocampus at low and high magnifications. Scale bar indicates 200 μm. (**b**) Neuronal density in the hippocampus was not significantly different among groups in all analyzed regions of the hippocampus and both ears. DG, dentate gyrus.

Hippocampal neurons are critical for cognition, memory, and learning, and have been shown to be vulnerable to various brain injuries^17,18^. To investigate neuronal density in the hippocampus after ear occlusion, Nissl staining of brain sections was performed (n = 5 animals per group; Fig. 2). Principal neurons in the hippocampus were counted, including neuronal cells in all subregions of the hippocampus, pyramidal cells in the CA1 and CA2/3 regions, and granule cells in the dentate gyrus (DG). Two-way ANOVA was performed for neuronal cell counts against factors of ear occlusion and laterality in the CA1, CA2/3, and DG subfields. No significant impact of ear occlusion or laterality was observed on neuronal density in the CA1, CA2/3, or DG subfields (CA1, *F*_*(2, 24)*_ = 0.01,* p* = 0.99 for ear occlusion and *F*_*(1, 24)*_ = 0.32,* p* = 0.57 for laterality; CA2/3, *F*_*(2, 24)*_ = 1.06,* p* = 0.36 for ear occlusion and *F*_*(1, 24)*_ = 2.07, *p* = 0.16 for laterality; DG, *F*_*(2, 24)*_ = 1.14, *p* = 0.34 for ear occlusion and *F*_*(1, 24)*_ = 0.70, *p* = 0.41 for laterality). Thus, suggesting that auditory deprivation in both uCHL and bCHL mice did not impact survival of hippocampal neurons.

### Neurogenesis in the hippocampus

**Figure 3:**
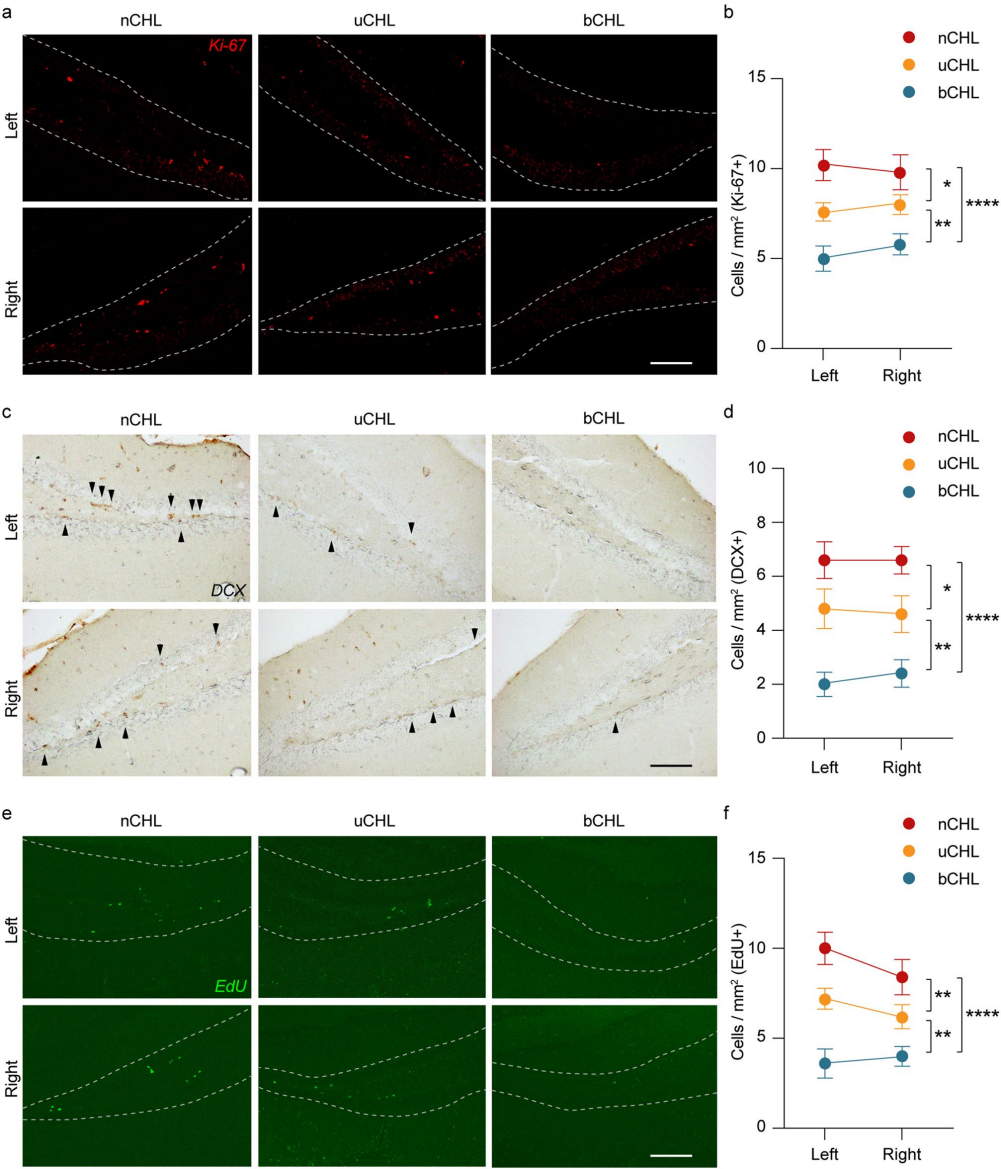
Auditory deprivation affected cell proliferation in the DG of the hippocampus. (**a**,**c**,**e**) Representative images of the hippocampus from each group of mice, immunostained for Ki-67, DCX, or EdU, five weeks after ear occlusion. Positive-staining cells were detected in the DG region of the hippocampus. (**b**,**d**,**f**) Summary of results regarding the number of Ki-67-positive, DCX-positive, and EdU-positive cells within the GCL. Significant differences among groups were observed. No significant differences between the ipsilateral and contralateral DG were detected across groups. The white dotted line indicates the border of the GCL. Scale bar indicates 100 μm. *p < 0.05, **p < 0.01, ****p < 0.0001. DG, dentate gyrus; GCL, granule cell layer.

Newly generated neurons can be detected in the DG of the hippocampus. Decreased neurogenesis has been reported to occur in the hippocampus following cochlear insult^11^. To evaluate the impact of auditory deprivation on neurogenesis, we stained the DG region of the hippocampus for Ki-67 (a marker of proliferation) and doublecortin (DCX; a marker of newly generated neurons within the last 2–3 weeks) after unilateral and bilateral CHL and counted the number of newly generated cells. Representative images of both Ki-67 (Fig. 3a) and DCX (Fig. 3c) staining indicated that Ki-67-positive and DCX-positive cells could be detected in the DG region of nCHL mice. A decrease in expression of neurogenesis markers was equally observed in both the right and left hippocampus following auditory deprivation, even for mice that had undergone unilateral CHL. The average numbers of Ki-67 and DCX-positive cells in both the ipsilateral and contralateral sides of the DG region are shown in Fig. 3b,d. We performed two-way ANOVA for both Ki-67-positive and DCX-positive cell counts against the factors of ear occlusion and laterality. Ear occlusion had a significant effect on the number of both Ki-67 and DCX cells (*F*_*(2, 24)*_ = 18.1*, p* < 0.0001 for Ki-67-positive cell counts and *F*_*(2, 24)*_ = 26.8,* p* < 0.001 for DCX-positive cell counts). However, ear occlusion did not have a significant effect on laterality (*F*_*(1, 24)*_ = 0.28, *p* = 0.60 for Ki-67 cells and *F*_*(1, 24)*_ = 0.02, *p* = 0.89 for Ki-67 cell counts). The number of Ki-67-positive cells in both bCHL and uCHL mice was significantly lower than that in nCHL mice (two-way ANOVA followed by post hoc analysis, nCHL vs. bCHL, *p* < 0.0001; nCHL vs. uCHL, *p* = 0.01; Fig. 3b). Furthermore, the number of Ki-67-positive cells was significantly lower in bCHL mice compared to that in uCHL mice (two-way ANOVA, uCHL vs. bCHL, *p* = 0.007). Similarly, the number of DCX-positive cells was significantly lower in bCHL and uCHL mice than in nCHL mice (two-way ANOVA, nCHL vs. bCHL, *p* < 0.0001; nCHL vs. uCHL, *p* < 0.01; Fig. 3d). In addition, the number of DCX-positive cells in bCHL mice was significantly lower than in uCHL mice (two-way ANOVA, uCHL vs. bCHL, *p* = 0.001).

To further address cell proliferation in the hippocampus, 5-Ethynyl-2′-deoxyuridine (EdU) was intraperitoneally injected into the mice 24 h before the sacrifice at a dose of 10 mg/kg to label newly generated cells. As shown in the representative photomicrographs in Fig. 3e, EdU-positive cells could be detected in the DG of the hippocampus of nCHL mice, similar to Ki-67-positive and DCX-positive cells. Decreased numbers of EdU-positive cells were observed equally in both the right and left hippocampus following auditory deprivation, even in uCHL mice. Figure 3f shows the average numbers of EdU-positive cells in both sides of the DG region. Two-way ANOVA was performed for evaluation of EdU-positive cell counts against the factors of ear occlusion and laterality. Ear occlusion had a significant effect on the number of EdU-positive cells (*F*_*(2, 24)*_ = 25.0,* p* < 0.0001), but laterality did not (*F*_*(1, 24)*_ = 1.38, *p* = 0.25). A significant decrease in the number of EdU-positive cells was observed in both bCHL and uCHL mice compared to nCHL mice (two-way ANOVA followed by post hoc analysis, nCHL vs. bCHL, *p* < 0.0001; nCHL vs. uCHL, *p* = 0.009). The number of EdU-positive cells in bCHL mice was lower than in uCHL mice (two-way ANOVA, uCHL vs. bCHL, *p* = 0.002). These results suggested that auditory deprivation, even in uCHL mice, could lead to impaired hippocampal neurogenesis and that bCHL dramatically decreased hippocampal neurogenesis even more so than uCHL.

### Activated microglia in the hippocampus

**Figure 4:**
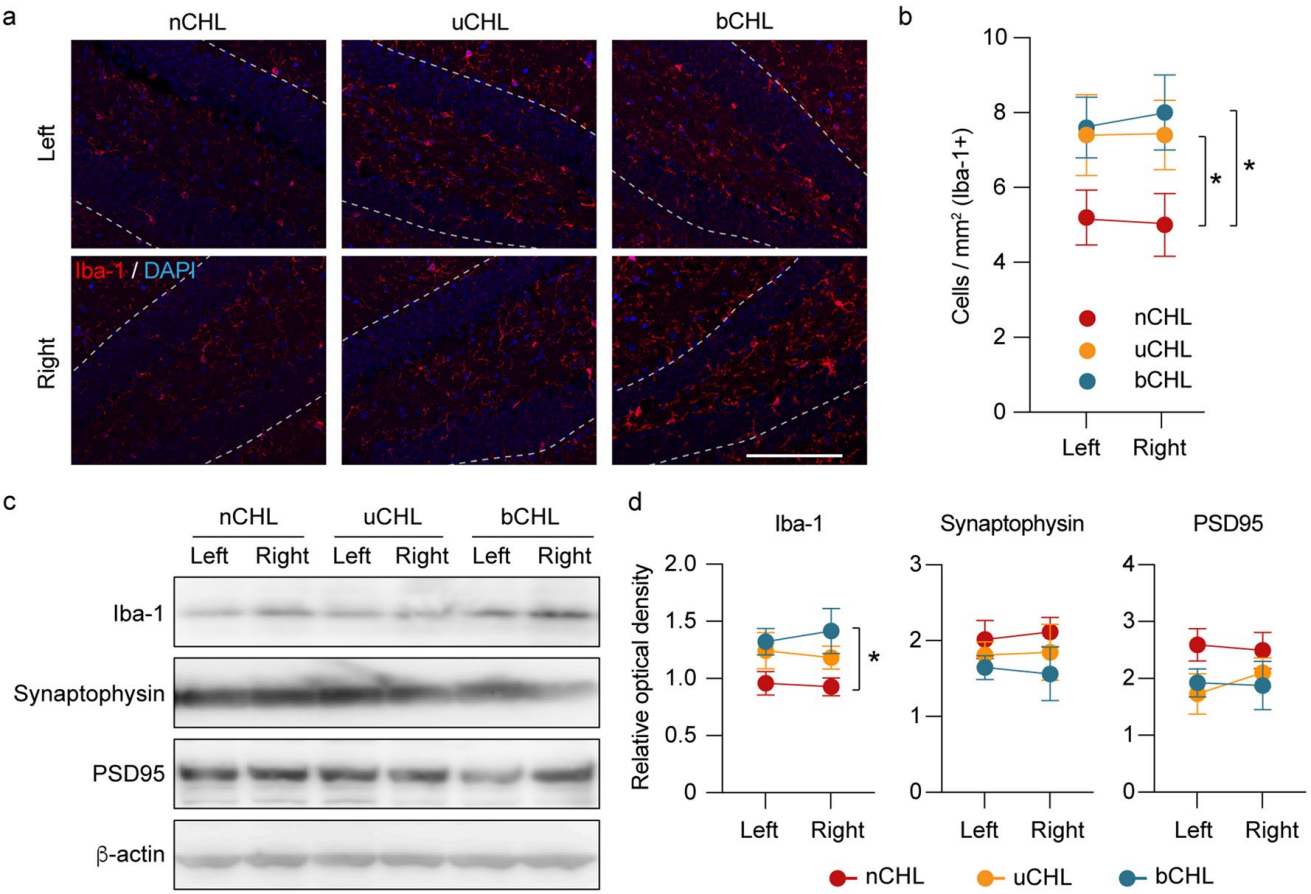
Iba-1 immunohistochemistry of the hippocampus and western blot analysis one week after ear occlusion. (**a**) Representative micrographs of hippocampus in DG stained for Iba-1 one week after ear occlusion. Scale bar indicates 100 μm. (**b**) Significant differences in the number of Iba-1-positive cells in both ears among the groups of mice were observed. No significant differences were detected between the right and left DG across groups. (**c**) Representative western blots for Iba-1, synaptophysin, and PSD95 one week after ear occlusion. (**d**) The expression levels of Iba-1 in uCHL and bCHL mice were upregulated one week after ear occlusion. Specifically, expression levels of Iba-1 in bCHL mice were significantly upregulated (n = 3 for each group). β -actin served as control for protein loading. *p < 0.05. DG, dentate gyrus.

Inflammatory processes in the brain mediated by activation of resident microglia lead to decreased neurogenesis^5,6^. To confirm the presence of microglia in the hippocampus after auditory deprivation, one week after CHL, brain sections were stained for Iba-1, a major marker for resting and activated microglia. Iba-1 expression was observed in the hippocampus, as indicated in the representative images of Iba-1 staining shown in Fig. 4a. Following auditory deprivation, increased Iba-1 expression was detected equally in both the right and left DG of the hippocampus, even in uCHL mice. Figure 4b shows the average numbers of Iba-1-positive cells in the DG region of both hippocampal sides. We performed two-way ANOVA for the number of Iba-1-positive cells against the factors of ear occlusion and laterality. Ear occlusion had a significant effect on the number of Iba-1 cells (*F*_*(2, 24)*_ = 5.18,* p* = 0.014), but laterality did not (*F*_*(1, 24)*_ = 0.008, *p* = 0.93). The numbers of Iba-1-positive cells in bCHL and uCHL mice were significantly higher than in nCHL mice (two-way ANOVA followed by post hoc analysis, nCHL vs. bCHL, *p* = 0.017; nCHL vs. uCHL, *p* = 0.046; Fig. 4b). There was no significant difference in the number of Iba-1-positive cells in uCHL mice compared to that in bCHL mice (two-way ANOVA followed by post hoc analysis, uCHL vs. bCHL, *p* = 0.90). Additionally, quantitative analysis for Iba-1 expression was performed by western blot showing increased Iba-1 expression in bCHL mice equally in both the right and left DG of the hippocampus (Fig. 4c). Further, we performed two-way ANOVA for Iba-1 expression against the factors of ear occlusion and laterality. Ear occlusion had a significant effect on Iba-1 expression (*F*_*(2, 12)*_ = 5.39,* p* = 0.021), but laterality did not (*p* = 0.99). The expression of Iba-1 in bCHL mice was significantly higher than in nCHL mice (two-way ANOVA followed by post hoc analysis, nCHL vs. bCHL, *p* = 0.021; Fig. 4d).

### Synaptic plasticity in the hippocampus

Synaptic plasticity has a central role in learning and memory and loss of synaptic vesicle proteins in the hippocampus correlates with cognitive decline^19^. To further address synaptic plasticity in the hippocampus after auditory deprivation, we evaluated the expression of the synaptic markers, synaptophysin and postsynaptic density protein 95 (PSD95) in the hippocampus after establishing the CHL model (Fig. 4c,d). Synaptophysin, a synaptic vesicle-associated protein commonly used as an estimate of the number of functional synapses, and PSD95, a pivotal postsynaptic scaffolding protein in excitatory neurons, are involved in the development and adjustment of the plasticity of nerve synapses^20^. Quantitative evaluation for synaptophysin and PSD95 expression was performed by western blot, but no significant differences of expression of these two synaptic markers were observed among groups, or between the right and left hippocampus. However, a downward trend could be observed in uCHL and bCHL mice (Fig. 4c,d), which might indicate that auditory deprivation could lead to hippocampal impairment of synaptic plasticity besides neurogenesis.

### Serum cortisol concentration

**Figure 5:**
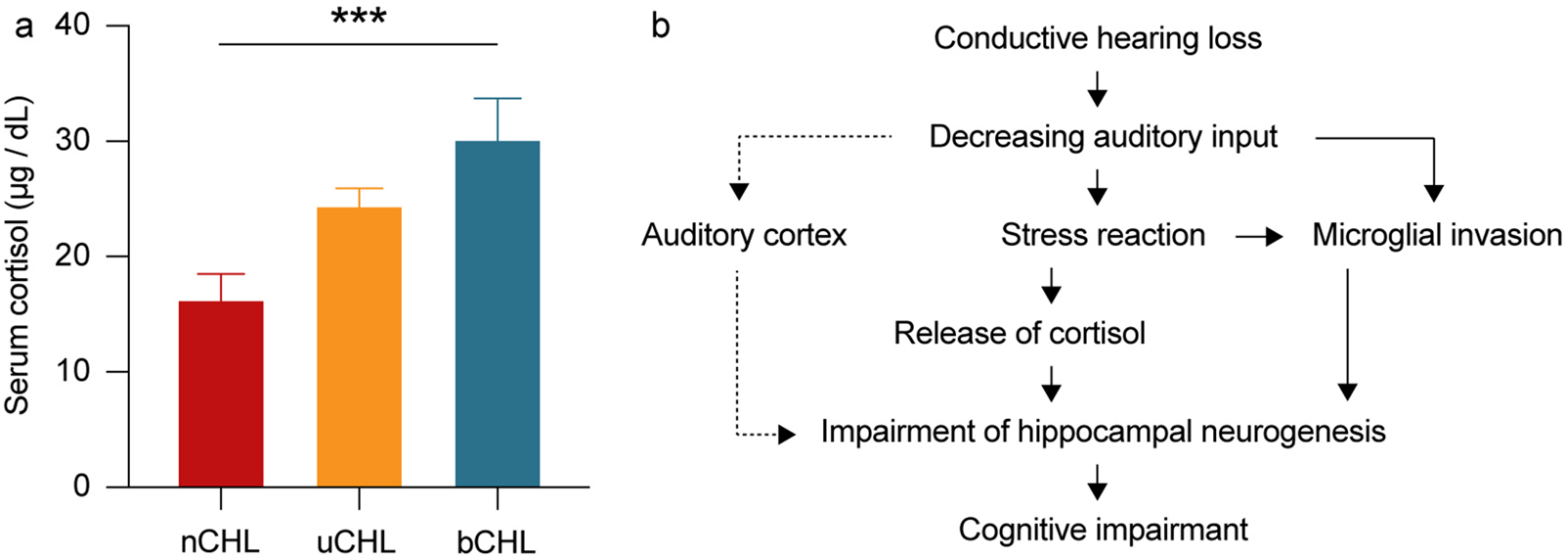
Serum cortisol concentrations one week after ear occlusion (**a**) and proposed mechanisms of hippocampal neurogenesis impairment following CHL (**b**). (**a**) Concentrations of serum cortisol were significantly higher in the bCHL group compared to that in the nCHL group. There were no significant differences between the nCHL and uCHL groups or the uCHL and bCHL groups (n = 5 per each group). ***p < 0.001. DG, dentate gyrus.

Hippocampal neurogenesis is known to be affected by stress responses^21^. Exposure to stress increases the release of glucocorticoid hormones, resulting in changes in hippocampal neurogenesis, considered to be one of the causes of mental disorders^22^. In addition, cortisol is considered a valid stress marker^23^. To investigate glucocorticoid hormone levels following auditory deprivation, we evaluated serum cortisol concentrations one week after ear occlusion (Fig. 5a). A significant difference was detected in cortisol concentrations among groups (one-way ANOVA, *p* = 0.0009) with cortisol levels increasing from nCHL mice, to uCHL mice, to bCHL mice. Serum cortisol concentrations were significantly higher in bCHL mice compared to that in nCHL mice (two-way ANOVA followed by post hoc analysis, nCHL vs. bCHL, *p* = 0.007). However, no significant differences were observed between nCHL and uCHL mice or uCHL and bCHL mice (two-way ANOVA, nCHL vs. uCHL, *p* = 0.07; uCHL vs. bCHL, *p* > 0.99). Thus, cortisol concentration significantly changed after ear occlusion, suggesting that stress progressively increased from nCHL mice to uCHL mice to bCHL mice.

## Discussion

In the current study, decreased auditory inputs following CHL induced significant impairment of hippocampal neurogenesis, despite no significant loss of SGNs or hippocampus neurons. Surprisingly, not only bCHL but uCHL also impaired neurogenesis and microglial invasion in the hippocampus. On average, the level of neurogenesis was reduced by almost 30% in uCHL mice and by nearly 50% in bCHL mice at 5-weeks post auditory deprivation. Interestingly, no significant difference was detected between the ipsilateral and contralateral sides of the hippocampus in uCHL mice. Furthermore, bCHL mice exhibited significantly less hippocampal neurogenesis in both sides of their hippocampus compared to uCHL mice. Our findings indicate that auditory activity had an essential role in regulating neurogenesis in the hippocampus. This suggests that typical neurogenesis impairment after cochlear insult may not be due only to the primary neuronal damage in the hippocampus, but also to decreased levels of the input sound. Furthermore, microglial invasion was equally and significantly increased in both the ipsilateral and contralateral hippocampus following uCHL, indicating that auditory deprivation, even unilaterally, could lead to microglial invasion. This microglial invasion might impair hippocampal neurogenesis, resulting in cognitive decline.

Neurogenesis is clearly associated with the pathophysiology of acute brain injury^24–26^, as well as neurodegenerative diseases such as Parkinson’s disease and Alzheimer's disease^27,28^. The impact of noise overexposure on cognitive function and hippocampal neurogenesis has been previously described^29^. Noise overexposure can suppress cell proliferation in the hippocampus, indicating impaired neurogenesis, consistent with our results of significantly reduced numbers of DCX-positive new neurons and Ki-67-positive and EdU-positive proliferating cells following CHL. Although noise overexposure causes morphological changes in the hippocampus^30^, in our current study, auditory deprivation in mice showed no quantitative neuronal loss, or synaptic plasticity in the hippocampus nor SGN loss. Therefore, neuronal death and synaptic disruption in the hippocampus was not necessarily required for the impairment of hippocampal neurogenesis. Moreover, noise exposure can cause both acute and long-term hyperactivity in the auditory system^31,32^. Auditory inputs are transmitted to the hippocampus through the auditory cortex and lemniscal ascending pathway^33^. A previous study showed that synaptic and biophysical mechanisms were disrupted at the level of the auditory cortex after CHL, and that a reduced potassium or increased calcium conductance in the auditory cortex after HL may underlie distorted membrane properties and imprecise timing^13^. Since the hippocampus responds to auditory stimuli, noise overexposure might lead to hyperactivity not only in the auditory system but also in the hippocampus^34^. Therefore, hippocampal neurogenesis might be down-regulated through this neural hyperactivity^35^. Furthermore, it has been reported that changing auditory input, especially decreasing it, results in hippocampal degeneration, spatial memory deterioration, and suppression of neurogenesis after cochlear insults^10^. Considering all of the above, the impaired neurogenesis observed in our study may relate to changes in the auditory pathway, especially to the decreasing input to the brain.

Microglia are a key element of hippocampal neurogenesis and coordinate inflammatory responses in the brain^4^. In addition, microglial activation contributes to decreased neurogenesis in the hippocampus^5,6^. Furthermore, in response to neuronal injury, microglia concentrate at the site of injury and are rapidly activated. This cellular response may play an important role in promoting the remodeling of affected neuronal circuits^36^. Within the auditory system, decreased auditory inputs result in reactive microglial and astrocytic responses in the central auditory pathway^37^. However, despite the increasing evidence suggesting microglial involvement in auditory function, none of these studies addressed microglial cell activation in the hippocampus following CHL. In the current study, we demonstrated that microglial invasion was equally and significantly increased in both the ipsilateral and contralateral hippocampus following either uCHL or bCHL. In the process of hippocampus neurogenesis, microglial invasion is activated by stress responses that lead to impairments in hippocampal neurogenesis^38^. Therefore, our results suggested that microglial invasion by CHL-induced stress resulted in the impairment of hippocampal neurogenesis (Fig. 5b), indicating that decreased auditory inputs caused by auditory deprivation might lead to microglial invasion, in turn influencing hippocampal neurogenesis and result in cognitive decline. Furthermore, a previous study showed that synaptic plasticity also played an important role in hippocampal functions such as learning and memory^39^. In the current study, we did not observe significant synaptic disruption in the hippocampus after auditory deprivation. Further research will be necessary to elucidate the relationship between microglial activation and synaptic plasticity followed by HL and the impairment of hippocampal neurogenesis.

Stress hormones are considered to have growth-inhibiting effects on various peripheral tissues and to inhibit cell proliferation, resulting in impaired hippocampal neurogenesis^40^. Consistent with this and our current results regarding hippocampal neurogenesis and microglia, we observed that cortisol levels increased following CHL, even in uCHL mice. However, how long this stress response may last following CHL and its actual relationship with neurogenesis and microglial invasion remains unclear.

The correlation between microglial activation, stress responses, and hippocampal neurogenesis following auditory deprivation is complicated. The hippocampus is the most vulnerable brain region under stress or other pathological conditions, and there is increasing evidence that acute psychological stress can trigger hippocampal inflammation, resulting in microglial activation and brain inflammatory cytokines^41,42^. In this study, we observed an increased stress reaction and microglial invasion one week after auditory deprivation. This result indicated that auditory deprivation would induce an acute stress reaction and microglial activation (Fig. 5b). The microglial inhibitor minocycline has been reported to block stress-induced neuroinflammation, a possible evidence that microglia play a central role in mediating the neuroinflammatory effects of stress^6^. Together, these findings suggest that the microglial invasion resulting from auditory deprivation-induced stress is critically involved in the suppression of neurogenesis. Moreover, inhibition of stress reactions following CHL might prevent the impairment of hippocampal neurogenesis by blockage of microglial invasion. The relationship between microglial activation, stress responses, and impairment of hippocampal neurogenesis after CHL warrants further investigation, however.

Globally, there are a large number of patients with HL, including SHL and CHL. Various treatment options, such as medication, surgery, and hearing aids, have been widely adopted to restore hearing function in these patients. The most commonly used devices for treating SHL are hearing aids, as SHL cannot be surgically treated^43^. In contrast, surgical treatment for patients with CHL may help in reducing the severity of the hearing impairment. However, not all patients receive medical treatment, especially those with unilateral HL, as they compensate by maximizing use of the better ear and minimizing use of the worse ear. A recent study revealed that only 11% of patients with unilateral HL wore hearing aids^44^. Furthermore, it has been clinically reported that unilateral childhood HL not treated with hearing aids results in lower performance at school and on intelligence tests^45^. However, whether unilateral HL also affects adults in a similar manner, resulting in decreased performance in learning, memory, and cognition, is not yet clear. Nor is it known why unilateral HL causes such poor outcomes, even though good hearing in the opposite ear remains. Our current results showed that uCHL in adult mice could lead to significant impairment of hippocampal neurogenesis, suggesting that decreased auditory input is a risk factor for the development of cognitive decline, even in unilateral HL. Given the minimal risk of hearing aid use and the potential benefits for patients with HL, such as preserved cognitive ability and improved hearing quality, we believe that public education regarding the complications and treatment of unilateral HL should be a priority. Patients with HL, even unilateral HL, should receive treatment to solve the hearing impairment, as well as to prevent cognitive decline.

In this study, we provide evidence that decreased sound levels lead to neurogenesis impairment, which is associated with cognitive decline, regardless of the presence of cochlear or hippocampal neuronal damage. Our results indicate that increasing sound levels with surgical treatment or the use of hearing aids may be reasonable, not only for the recovery of hearing thresholds but also for preventing or improving cognitive impairment. As such, this study identifies significant implications for the clinical treatment of patients with HL. Furthermore, our auditory deprivation model should be useful to more deeply investigate the mechanisms of hippocampal and auditory impairment. However, limitations of the study should also be acknowledged. First, while we demonstrated that following CHL, decreased hippocampal neurogenesis resulted in mice, we did not assess the animals’ cognitive function using behavioral tests. Additional research will be necessary to evaluate behavioral tests for cognitive, learning, and memory functions in CHL mice. Second, the observation period of our study following CHL was relatively short. Therefore, we are unable to determine how the neurogenesis impairment continues with respect to long-term CHL. Third, the mice used in this study included only young adult mice. The effects of auditory deprivation on hippocampal neurogenesis might differ in an age-dependent manner. Future research with a long-term follow-up of CHL in various aged mice will be required to elucidate the full capacity of neurogenesis.

## Conclusions

The findings of this study are expected to contribute toward clarifying the mechanisms of cognitive decline associated with HL. Decreased auditory input, as a result of auditory deprivation, significantly impaired hippocampal neurogenesis, even in uCHL mice, despite no significant hippocampal neuronal loss. In addition, bCHL exhibited a significant neurogenesis impairment and increased microglial invasion compared to nCHL and uCHL. Furthermore, bCHL showed a significant increase in stress responses compared to nCHL. Therefore, preventing decreased auditory input in patients with HL might facilitate hippocampal neurogenesis.

## Methods

### Animals and ear occlusion

All animal experiments were performed in accordance with the guidelines of the Animal Experimentation and Ethics Committee of Kitasato University School of Medicine. For this study, 8-week-old male C57BL/6 mice were used to perform the ear occlusions. Mice were intraperitoneally injected with medetomidine (0.75 mg/kg), midazolam (4 mg/kg), and butorphanol (5 mg/kg), and a post-auricular incision was performed. The external auditory canal was identified and transected. To close the ear canal, an ear mold silicone compound was plugged into the ear canal followed by ligation of the distal portion of the external auditory canal with 4–0 surgical silk suture. Animals were evaluated every week to ensure that the ear occlusions remained, and divided into three groups (Fig. 1a). Stress and inflammatory reaction such as immune cytokine release occur in days after exposure to stress^46^. Therefore, five mice per each group were sacrificed at nine weeks of age (one week post occlusion) for the assessment of microglial expression and serum cortisol level. Newborn neurons in the hippocampus express DCX for approximately 2–3 weeks after they are born and noise-induced hearing loss leads to hippocampal impairment of neurogenesis 1–3 month after noise exposure^11^. Therefore, eight mice per each group were sacrificed at 13-weeks of age (5-weeks post occlusion) for the assessment of cochlear and brain tissue. The cochleae were examined for SGN enumeration, and the brains were examined for Nissl staining and used for immunohistochemical analysis.

### Auditory brainstem response

We measured ABR to determine hearing thresholds, as previously described^16^. Briefly, a total of 256 ABRs were averaged and recorded at 5-dB sound pressure level intervals (Nihon Koden, Tokyo, Japan). ABRs were measured prior to ear occlusion at 8-weeks of age and 5-weeks after occlusion at 13-weeks of age (n = 8 animals per group; Fig. 1a).

### SGN analysis

To quantitatively assess the SGNs, plastic sections were prepared as described previously^47^. The tissues were sectioned using a glass knife and Leica UC7 microtome, and the sections stained with toluidine blue. The sections were observed and the images captured using an Olympus BX53 microscope (Olympus, Tokyo, Japan) with an Olympus DP27 camera. To calculate SGN density, the number of SGNs in the middle turn of the cochlea were counted, and the area of the Rosenthal’s canal measured for three sections per animal (n = 3 animals per group).

### Brain tissue preparation

After intracardial perfusion with 4% paraformaldehyde (PFA) in phosphate buffer (PB), brainstems were removed and then postfixed overnight in 4% PFA. The brainstems were then cryoprotected in 30% sucrose and frozen using liquid nitrogen. Transverse brain Sects. (20 µm) were prepared from the frozen specimens and mounted onto glass slides. Cresyl violet was used for Nissl staining and stereological analysis of the hippocampus. Images were captured using an Olympus BX53 microscope with an Olympus DP27 camera. The density of hippocampal neurons in CA1, CA2/3, and DG was measured using ImageJ software (NIH, https://imagej.nih.gov/ij/download.html). The number of hippocampal neurons was quantified in four sections per animal (n = 5 animals) at equal intervals from the caudal to rostral regions for assessment of neuronal density in CA1, CA2/3, and DG.

### Immunocytochemistry and hippocampal evaluation

Immunohistochemical staining of Ki-67 and Iba-1 was performed using an immunofluorescent technique. Brain sections were incubated for 30 min in 1% normal goat serum (NGS) in phosphate buffer saline (PBS) containing 0.3% Triton X-100 for blocking, followed by incubation overnight with primary antibodies anti-Iba-1 (rabbit, 1:400, Wako). After washing in PBS, sections were incubated with goat-anti rabbit secondary antibody (Molecular Probes, Eugene, OR) for one hour. After washing, 4′,6-diamidino-2-phenylindole (DAPI) was used to counterstain the DNA in the Iba-1 stained sections, and slides were cover-slipped using VECTASHIELD mounting medium (Vector Laboratories, Burlingame, CA, USA). The immunostained sections were observed under a confocal laser microscope (LSM710 microscope, Zeiss, Jena, Germany).

The 3,3′-diaminobenzidine (DAB) technique was used for DCX immunohistochemical staining. Brain sections were treated with 0.6% H_2_O_2_ and then permeabilized with 0.25% Triton X-100 in PBS. After washing, sections were blocked with 2.5% NGS in PBS for two hours followed by overnight incubation with anti-DCX primary antibody (mouse, 1:50, Santa Cruz). After extensive washing, sections were incubated with biotinylated secondary antibodies for one hour. Immunoreactivity was visualized using DAB as substrate. Sections were dehydrated with alcohol, clarified with xylene, and cover-slipped using Permount (Thermo Fisher Scientific). The stained sections were observed using an Olympus BX53 microscope with an Olympus DP27 camera.

Cell proliferation was evaluated using a Click-iT EdU Alexa Fluor 488 Imaging Kit (Thermo Fisher Scientific). Briefly, EdU was intraperitoneally injected into the mice 24 h before the sacrifice at a dose of 10 mg/kg. EdU staining was performed following manufacturer’s instructions. Slides containing hippocampal tissue were observed under a confocal laser microscope (LSM710 microscope).

For cell counting, four pictures of the hippocampus were obtained at equal intervals from caudal to rostral. Positive cells (Ki67, Iba-1, DCX, and EdU) in the DG were counted in four sections for each animal from the left and right side of the hippocampus. For Ki-67, DCX, and EdU, the number of positive-staining cells was divided by the measured length of the DG. For Iba-1, the number of positive-staining cells was divided by the measured area of the DG, specifically in the subgranular zone. To reduce potential counting bias, counting procedures were performed blinded as to whether the tissue was from normal or CHL mice.

### Western blot

The hippocampus was removed and homogenized in RIPA buffer with protease inhibitor cocktail (Nacalai tesque). The homogenate was centrifuged at 15,000 rpm for 10 min at 4 °C. The supernatants were separated by sodium dodecyl sulfate polyacrylamide gel electrophoresis (e-PAGEL-HR, ATTO), and proteins transferred onto an Immobilon-P membrane (Clear Blot Membrane-P plus, ATTO). The membranes were blotted with antibodies of anti-Iba-1 (019–19,741, rabbit, 1:500, Wako), anti-synaptophysin (ab32127, rabbit, 1:1000, Abcam), anti-PSD95 (ab18258, rabbit, 1:10,000, Abcam) and anti-β-actin (G043, mouse, 1:1000, Abm) antibodies. The sections were treated with secondary antibody (NA931 and NA934, 1:10,000, GE Healthcare) and protein bands visualized with a chemiluminescence detection system (ECL Select Western Blotting Detection Reagent, Amersham). Immunoblot signals were analyzed by a LAS-4000 digital imaging system (Fujifilm, Tokyo, Japan). Band intensities were quantified and divided by their corresponding loading controls (β-actin) (n = 3 animals per group).

### Cortisol measurement

Blood samples were collected by cardiac puncture one week after ear occlusion just prior to the sacrifice, and sera prepared. Serum cortisol levels were determined using a Cortisol Enzyme-Linked Immunosorbent Assay (ELISA) Kit (ADI-900–071, ENZO) according to the manufacturers’ protocols. Concentrations of cortisol were calculated by comparing the samples to standard curves generated using reagents provided with the kit.

### Statistical analyses

GraphPad Prism 8.2.1 (Graphpad Software Inc., La Jolla, CA) was used for statistical analyses. The normality of the data was first verified using the Shapiro–Wilk test. For normally distributed data, a one-way or two-way ANOVA was used to evaluate differences between groups with correction for multiple comparisons using Tukey’s post hoc test. For non-normally distributed data, the non-parametric Kruskal–Wallis test followed by Dunn’s multiple comparison test was used. ABR results were evaluated using two-way ANOVA followed by Tukey’s multiple comparisons test. All data are presented as means ± standard error. A p-value < 0.05 was considered statistically significant and reported as follows: *p < 0.05; **p < 0.01; ***p < 0.001; and ****p < 0.0001.

### Ethical approval

All procedures performed in studies involving animals were in accordance with the ethical standards of the Ethics Committee of the Kitasato University School of Medicine (2019–085).

## Supplementary information

**Supplementary Figures**

## Abbreviations

ABR: Auditory brainstem response
CHL: Conductive hearing loss
DG: Dentate gyrus
EDU: 5-Ethynyl-2′-deoxyuridine
GCL: Granule cell layer
HL: Hearing loss
NGS: Normal goat serum
PB: Phosphate buffer
PBS: Phosphate buffered saline
PFA: Paraformaldehyde
SGN: Spiral ganglion neuron
SHL: Sensorineural hearing loss
