# Supplementary material for: Decreasing auditory input induces neurogenesis impairment in the hippocampus
Source: Sci Rep. 2021 Jan 11;11:423. doi: 10.1038/s41598-020-80218-z (PMC7801596; doi:10.1038/s41598-020-80218-z)
Supplement: Supplementary file 1 — Supplementary Figures [file 41598_2020_80218_MOESM1_ESM.docx]

Decreasing auditory input induces neurogenesis impairment in the hippocampus

Takaomi Kurioka,* Sachiyo Mogi, and Taku Yamashita

**Supplementary Information**

**Figure.S1**


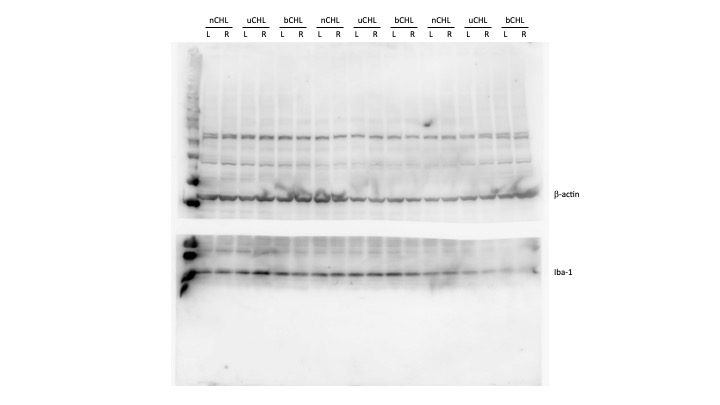
Western blot analysis of Iba-1. Two blots were exposed and captured in one image.

**Figure.S2**


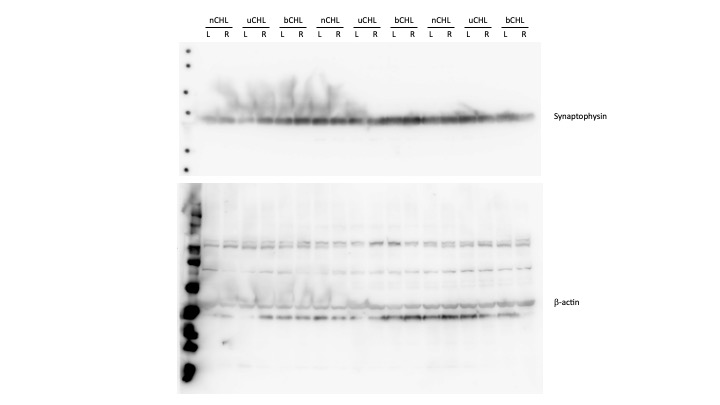
Western blot analysis of Synaptophysin. Two blots were exposed and captured in one image.

**Figure.S3**


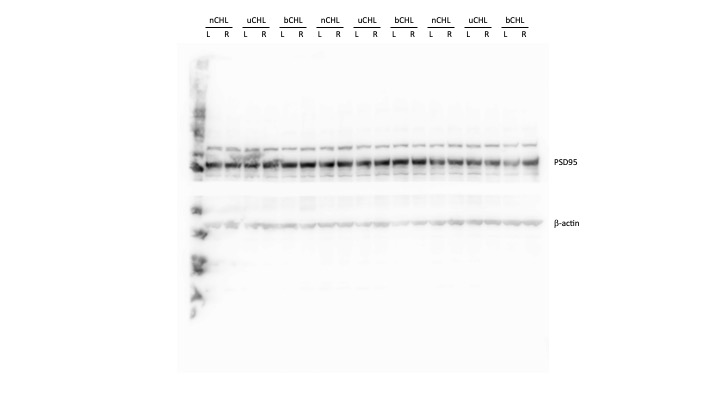
Western blot analysis of PSD95. Two blots were exposed and captured in one image.
